# US Nonprofit Hospitals’ Community Health Needs Assessments and Implementation Strategies in the Era of the Patient Protection and Affordable Care Act

**DOI:** 10.1001/jamanetworkopen.2021.22237

**Published:** 2021-08-24

**Authors:** Leo Lopez, Meera Dhodapkar, Cary P. Gross

**Affiliations:** 1 National Clinician Scholars Program, Department of Internal Medicine, Yale School of Medicine, New Haven, Connecticut; 2 Yale School of Medicine, New Haven, Connecticut; 3 Section of General Internal Medicine, Department of Internal Medicine, Yale School of Medicine, New Haven, Connecticut; 4 Cancer Outcomes Public Policy and Effectiveness Research Center, Yale School of Medicine, New Haven, Connecticut

## Abstract

This cross-sectional study examines the proportion of US nonprofit hospitals with community health needs assessments and implementation strategies as required by the Patient Protection and Affordable Care Act.

## Introduction

In response to congressional concerns that US nonprofit hospitals were providing insufficient community benefit to justify their tax-exempt status (estimated at $24.6 billion in 2011),^1^ the Patient Protection and Affordable Care Act (ACA) added new requirements. These rules mandated that all nonprofit hospitals (1) conduct a triennial community health needs assessment (CHNA) and adopt an implementation strategy, (2) abide by specific documentation requirements, and (3) make these documents publicly available. This cross-sectional study examines US nonprofit hospitals’ adherence to these requirements during the ACA era.

## Methods

The Yale University Human Research Protection Program determined that this study is not considered human participants research; thus, neither institutional review board approval nor informed consent was needed, in accordance with 45 CFR §46. This study follows the Strengthening the Reporting of Observational Studies in Epidemiology (STROBE) reporting guidelines for cross-sectional studies.

We used a standardized search on the ProPublica Nonprofit Explorer in January 2019 and identified 1662 nonprofit hospitals in the US and the District of Columbia.^2^ We randomly selected 500 of these hospitals, proportionally by the number of active nonprofit hospitals in each state in 2017. We then accessed their respective Internal Revenue Service form 990 Schedule H directly from ProPublica. From these forms, we assessed whether each hospital reported that it had conducted a CHNA, adopted an implementation strategy, and made these reports available online.

We downloaded the CHNAs and implementation strategies directly from hospitals’ websites. We used 7 specific Internal Revenue Service documentation elements to evaluate the available CHNAs and used 3 reporting requirements to evaluate the implementation strategies^3^ (Table 1). Similar to prior studies, we used a Likert scale to rate the level of detail of each CHNA and implementation strategy (range, 0-5, with higher scores indicating more detail and higher quality).^4^ A co-rater (L.L.) conducted a review of 10% of the sample using the same instrument, and the κ value was greater than 0.8. The analyses were conducted in Stata statistical software version 15.1 (StataCorp) in June 2020.

**Table 1.** CHNA and Implementation Strategy Documentation Elements as Required by the Internal Revenue Service

Documentation	Requirements
CHNA	A definition of the community served by the hospital facility and a description of how the community was determined
	A description of the process and methods used to conduct the CHNA
	A description of how the hospital facility solicited and took into account input received from persons who represent the broad interests of the community it serves
	A description of the medically underserved, low-income, or minority populations being represented by organizations or individuals that provided input
	A prioritized description of the significant health needs of the community identified through the CHNA
	A description of resources potentially available to address the significant health needs identified through the CHNA
	An evaluation of impact of any actions that were taken to address the significant health needs identified in the immediately preceding CHNA
Implementation strategy	The actions the hospital will take to address each health need identified in the CHNA
	The associated resources devoted to and the anticipated impact of each action taken
	Description of the planned collaborations between the hospital and other institutions to address the health need

Abbreviation: CHNA, community health needs assessment.

## Results

Among the 500 hospitals in our sample, 495 (99.0%) reported on their Internal Revenue Service 990 form that they had conducted a CHNA, and 412 (84.0%) of these CHNAs were identified online. A total of 491 hospitals (99.0%) reported that they adopted an implementation strategy, and 331 of these (75.0%) were identified on their website. In aggregate, 229 (60.0%) of the hospitals in our sample had both a CHNA and corresponding implementation strategy that could be found online.

The 412 CHNAs had a mean quality score of 3.2 of 5, consistent with partial detail. Many were missing the required documentation elements: 174 CHNAs (42.2%) did not include an evaluation of impact description, and 101 (25.0%) did not describe the resources available to address the health needs they identified (Table 2). The 331 implementation strategies had a mean quality score of 3.2 of 5; 136 (41.0%) were rated as solid-high quality (score 4 or 5 of 5).

**Table 2.** Quality Scores for CHNA Reports by Documentation Requirement

CHNA element	Score, mean	CHNAs, No. (%)			
		Solid-high quality by element (score 4/5 or 5/5)	Variable quality by element (score 3/5)	Low-weak quality by element (score 1/5 or 2/5)	Did not address element (score 0/5)
Definition of community	4.17	335 (81.3)	55 (13.4)	11 (2.86)	9 (2.2)
Methods	4.14	324 (79.0)	55 (13.4)	25 (6.1)	6 (1.5)
Input from community	3.71	292 (71.0)	77 (18.8)	24 (5.9)	16 (3.9)
Description of underserved population	2.39	167 (41.0)	51 (12.4)	43 (10.5)	149 (36.0)
Prioritization of health needs	2.8	126 (31.0)	148 (36.1)	89 (21.7)	47 (11.4)
Resources available	3.24	240 (58.0)	39 (9.5)	29 (7.1)	101 (25.0)
Evaluation of impact since last CHNA	2.13	172 (42.0)	34 (8.3)	29 (7.1)	174 (42.2)

Abbreviation: CHNA, community health needs assessment.

## Discussion

This cross-sectional study found that since the passage of the ACA’s CHNA and implementation strategy regulations, most hospitals reported that they are conducting CHNAs and adopting related implementation strategies. However, only 60.0% of the hospitals in our sample had both a CHNA report and an implementation strategy on their website, and many of the documents were missing the required documentation elements.

This study had some limitations. Our sampling methods were determined according to the ProPublica database. However, we are confident in the comprehensiveness of this source because it includes more than 3 million tax filings under 27 different nonprofit designations.^2^

The ACA sought to ensure that hospitals fulfill their obligations to their communities. However, many CHNAs and implementation strategies are not available at all, and those that are accessible do not provide the required information regarding how hospitals are assessing and addressing community health needs. There is much work to be done, and federal policy makers have an opportunity to improve hospitals’ accountability and transparency.

## References

[1] Nikpay SS, Ayanian JZ. Hospital charity care: effects of new community-benefit requirements. N Engl J Med. 2015;373(18):1687-1690. doi:10.1056/NEJMp1508605 26510018

[2] Schwencke K, Tigas M, Wei S, Glassford A, Suozzo A, Roberts B. Nonprofit explorer research tax-exempt organizations. ProPublica. Published December 16, 2019. Accessed January 3, 2019. https://projects.propublica.org/nonprofits/

[3] Internal Revenue Service. Community health needs assessment for charitable hospital organizations: section 501(r)(3). Published 2015. Accessed July 19, 2021. https://www.irs.gov/charities-non-profits/community-health-needs-assessment-for-charitable-hospital-organizations-section-501r3

[4] Pennel CL, McLeroy KR, Burdine JN, Matarrita-Cascante D. Nonprofit hospitals’ approach to community health needs assessment. Am J Public Health. 2015;105(3):e103-e113. doi:10.2105/AJPH.2014.302286 25602862 PMC4330850

